# Identification and screening of effective protective antigens for channel catfish against *Streptococcus iniae*

**DOI:** 10.18632/oncotarget.16475

**Published:** 2017-03-22

**Authors:** Yajun Wang, Erlong Wang, Yang He, Kaiyu Wang, Qian Yang, Jun Wang, Yi Geng, Defang Chen, Xiaoli Huang, Ping Ouyang, Weimin Lai, Cunbin Shi

**Affiliations:** ^1^ Department of Basic Veterinary, Sichuan Agricultural University, Chengdu, Sichuan, China; ^2^ Pearl River Fishery Research Institute, Chinese Academy of Fishery Science, Guangzhou, Guangdong, China; ^3^ Key Laboratory of Animal Disease and Human Health of Sichuan Province, Sichuan Agricultural University, Chengdu, Sichuan, China\; ^4^ Department of Aquaculture, Sichuan Agricultural University, Chengdu, Sichuan, China

**Keywords:** Streptococcus iniae, immunogens, immunogenicity, subunit vaccines, channel catfish, Immunology and Microbiology Section, Immune response, Immunity

## Abstract

Vaccination is a potential approach for prevention and control of disease in fish. The use of genetically engineered vaccines is an effective method and a green intervention to control bacterial infection in aquaculture. However, efforts to develop these vaccines are limited by the lack of conserved protective antigens. In this study, three candidate immunogens (Srr, NeuA, and Hsp) of the pathogenic *Streptococcus iniae* strain DGX07 isolated from diseased channel catfish were identified and analyzed. Molecular cloning, expression, and purification of candidate antigen genes were carried out to obtain the candidate immunogens in the form of recombinant subunit vaccines. Western blotting was performed to evaluate immunogenicity *in vitro* and channel catfish were vaccinated by intraperitoneal injection and the specific antibody titers and relative percent of survival were determined to evaluate immune protection *in vivo*. The results showed that these three candidate immunogens were expressed correctly as recombinant proteins fused with His tags, with molecular weights of 70 kDa for Srr, 86 kDa for NeuA, and 51 kDa for Hsp, respectively. Moreover, each immunogen was predicted to be located either extracellularly or on the surface of *S. iniae*, and were able to offer protection against *S. iniae* infection in the form of recombinant subunit vaccines with adjuvant ISA763, especially Srr, with a relative percent of survival of 70% for Srr, 55% for NeuA, and 50% for Hsp, respectively.

## INTRODUCTION

Channel catfish (*Ictalurus punctatus*) is a highly adaptive species extensively cultured worldwide that is the leading aquaculture species in China and accounts for more than 60% of aquaculture production in the United States [1]. Moreover, among ectothermic vertebrates in scientific research, this fish is widely used as a model for comparative immunology, reproductive physiology, and toxicology [2]. Unfortunately, the use of this model has been compromised in recent years by widespread disease outbreaks due to various bacterial pathogens [3–6] especially *Streptococcus iniae*, a Gram-positive β-hemolytic species, which is a major pathogen of farmed fish that causes severe damage and heavy economic losses to the fish farming industry. The most susceptible fish species include both marine and freshwater fish such as Japanese flounder [7, 8], European sea bass [9, 10], Asian seabass [11], rainbow trout [12, 13], zebrafish [14], Nile tilapia [14], and channel catfish [5, 16]. In addition, *S. iniae* is also known to be an opportunistic pathogen in humans [17, 18].

Streptococcal disease has been shown to occur by several routes which include ingestion of streptococcal contaminated fish food [19, 20], cohabitation and subsequent ingestion of moribund fish, injured skin prior to immersion in water contaminated with Streptococcus sp [21, 22]. It had been demonstrated that *S. iniae* could entry into the fish through the olfactory organ (i.e. nares inoculation) [20, 23], injured skin [21] and the gills [24]. Also, the routes of entry of *S. iniae* is different due to different species. In humans, Streptococcus sp. colonize the nasal mucosa and then become systemic causing meningitis [20]. The skin abrasion, gills, nasal mucosa and gastrointestinal (GI) tract are the portals for *S. iniae* to entry into the fish, which reported in tilapia and hybrid striped bass [20, 23, 24]. As for channel catfish, whether the routes of entry of *S. iniae* is similar to tilapia and hybrid striped bass or similar to another bacterial pathogen, *Edwardsiella ictaluri* via the gills [25] and the olfactory organ mucosa [26] need further investigation.

Vaccination is an effective green intervention, as opposed to the traditional use of antibiotics and antimicrobial compounds, to control *S. iniae* infection in aquaculture [16]. Compared with inactivated and/or attenuated live vaccines, genetically engineered vaccines, such as subunit vaccines and DNA vaccines composed of protective immunogens or antigen genes, are safer and more serotype-independent [27]. However, efforts to develop subunit vaccines are limited by the lack of the conserved protective antigens. Hence, the identification and screening of novel and conservative immunogenicity antigens is crucial for the development of an effective subunit vaccine [28].

In this study, three candidate immunogens, Srr, NeuA, and Hsp, of the pathogenic *S. iniae strain* DGX07 isolated from diseased channel catfish were identified and analyzed. *In vivo* and *in vivo* immunogenicity evaluations were conducted to determine whether these three candidate immunogens could be used as an effective vaccine against *S. iniae* infection of channel catfish. The results of this study showed that the immunoprotective efficacy of these three immunogens used as candidate vaccines was in the order of Srr > NeuA > Hsp.

## RESULTS

### PCR amplification, cloning, and sequence analysis of candidate antigen genes

**Figure 1:**
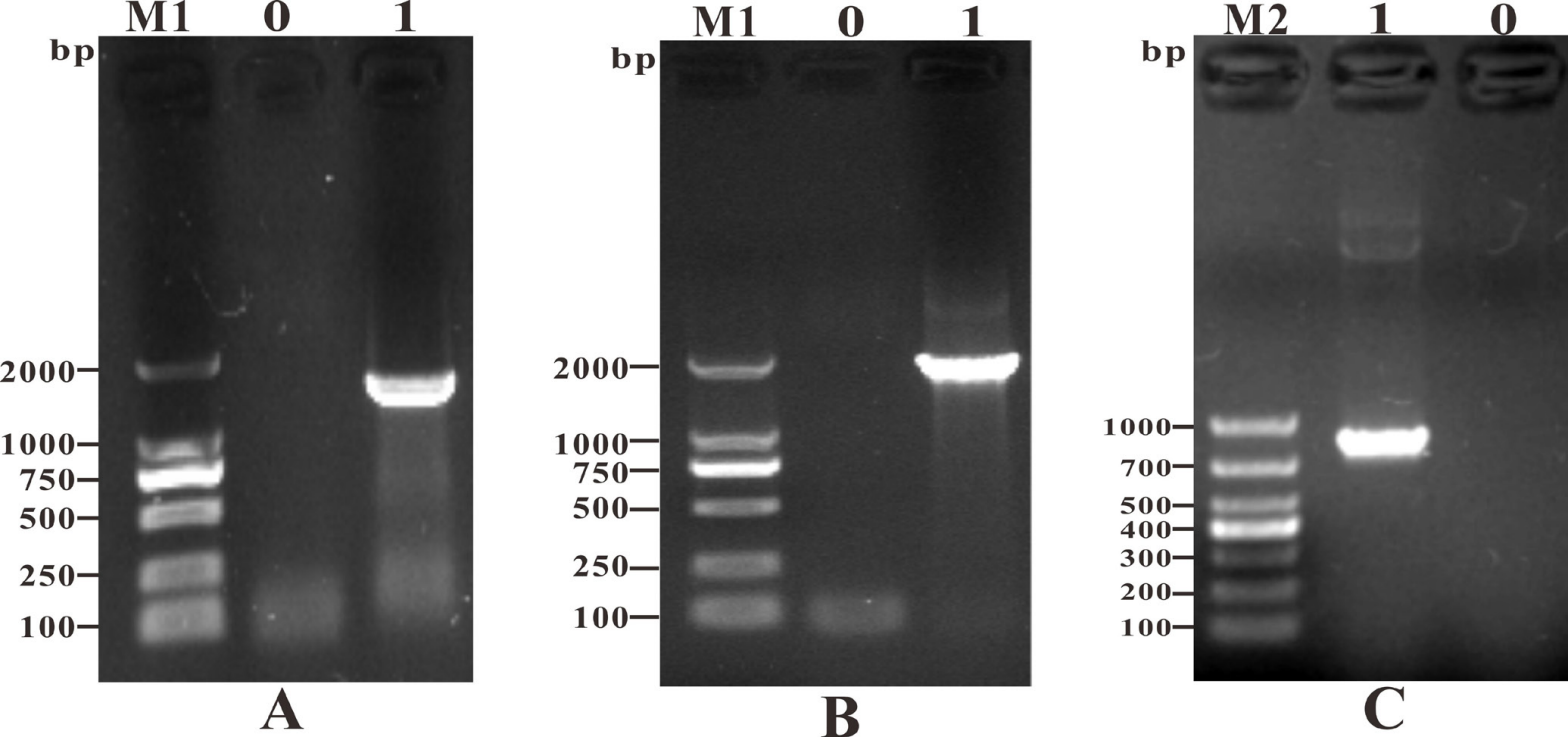
PCR amplification of candidate antigen genes **A**. M1: DNA Marker (DL2000); Lane 0: negative control; Lane 1: PCR product of the *Srr* gene (1320 bp); **B**. M1: DNA Marker (DL2000); Lane 0: negative control; Lane 1: PCR product of the *NeuA* gene (1833 bp); **C**. M2: DNA Marker (DL1000); Lane 0: negative control; Lane 1: PCR product of the *Hsp* gene (867 bp).

**Figure 2:**
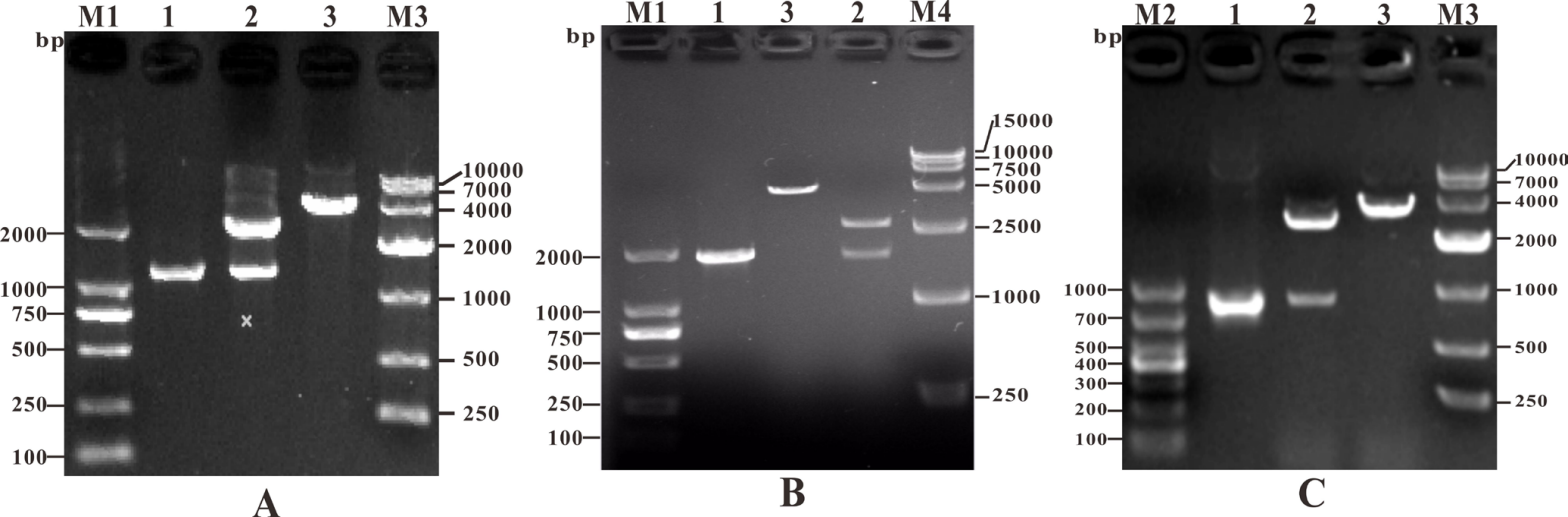
Identification of recombinant cloning plasmids by PCR and restriction enzyme digestion **A**. M1: DNA Marker (DL2000); Lane 1: PCR product of recombinant cloning plasmid T-*Srr*; Lane 2: digestion of plasmid T-*Srr* with *Bam*HI + *Xho*I; Lane 3: digestion of plasmid T-*Srr* with *Bam*HI; M3: DNA Marker (DL10000). **B**. M1: DNA Marker (DL2000); Lane 1: PCR product of recombinant cloning plasmid T-*NeuA*; Lane 2: digestion of plasmid T-*NeuA* with *Bam*HI + *Xho*I; Lane 3: digestion of plasmid T-*NeuA* with *Bam*HI; M4: DNA Marker (DL15000). **C**. M2: DNA Marker (DL1000); Lane 1: PCR product of recombinant cloning plasmid T-*Hsp*; Lane 2: digestion of plasmid T-*Hsp* with *Bam*HI + *Xho*I; Lane 3: digestion of plasmid T-*Hsp* with *Bam*HI; M3: DNA Marker (DL10000).

To obtain the candidate antigen genes, PCR was conducted with specific primers. The lengths of the PCR products *Srr, NeuA*, and *Hsp* were 1320 bp, 1833 bp, and 867 bp, respectively (Figure 1). The recombinant cloning plasmids (T-Srr, T-NeuA, and T-Hsp) were identified correctly by PCR, enzymatic digestion, and sequencing, and the target bands were consistent with the expected sizes (Figure 2). Then, the nucleotide sequences of the *Srr, NeuA*, and *Hsp* genes were submitted to the GenBank database (https://www.ncbi.nlm.nih.gov/genbank/) and assigned the accession numbers KY030834, KU925348, and KY030835, respectively.

**Figure 3:**
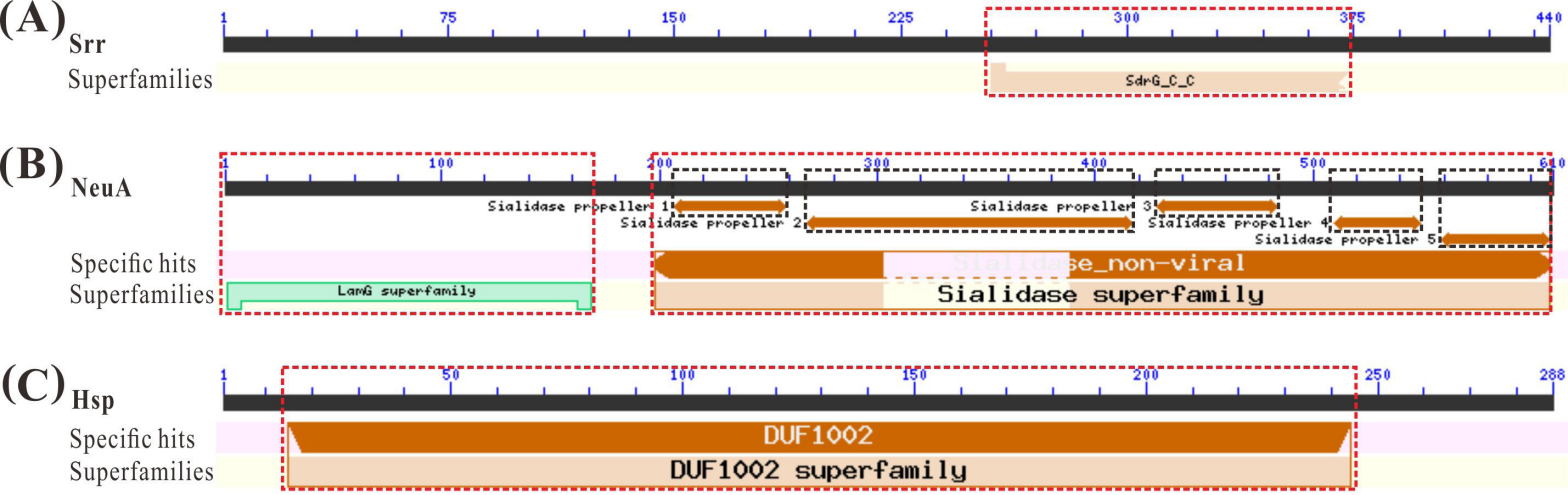
Conserved domain analysis of vaccine candidate antigens (**A**). Conserved domain analysis of candidate antigens Srr with a SdrG_C_C superfamily domain. (**B**). Conserved domain analysis of candidate antigens NeuA with a LamG superfamily domain and a Sialidase_non-viral domain belonging to the Sialidase superfamily. (**C**). Conserved domain analysis of candidate antigens Hsp with a DUF1002 superfamily domain.

The molecular weights and isoelectric points of the derived amino acids were predicted, respectively, as 47.99 kDa and 4.45 for Srr, 68.84 kDa and 6.67 for NeuA, and 31.14 kDa and 5.24 for Hsp using the ExPASy molecular biology server (http://www.expasy.org/tools). Analysis of the conserved domains showed that Srr contained a C-terminus of bacterial fibrinogen-binding adhesion domain at aa 255-373 belonging to SdrG_C_C superfamily. The NeuA contained a laminin G domain (aa 2-169) belonging to LamG superfamily and a non-viral sialidase domain (aa 198-609) which contained five sialidase propellers and belonged to the sialidase superfamiy, and Hsp contained a DUF1002 domain (aa 15-244) belonging to the DUF1002 superfamily (Figure 3).

### Multiple sequence alignment and phylogenetic analyses of candidate antigen genes

**Figure 4:**
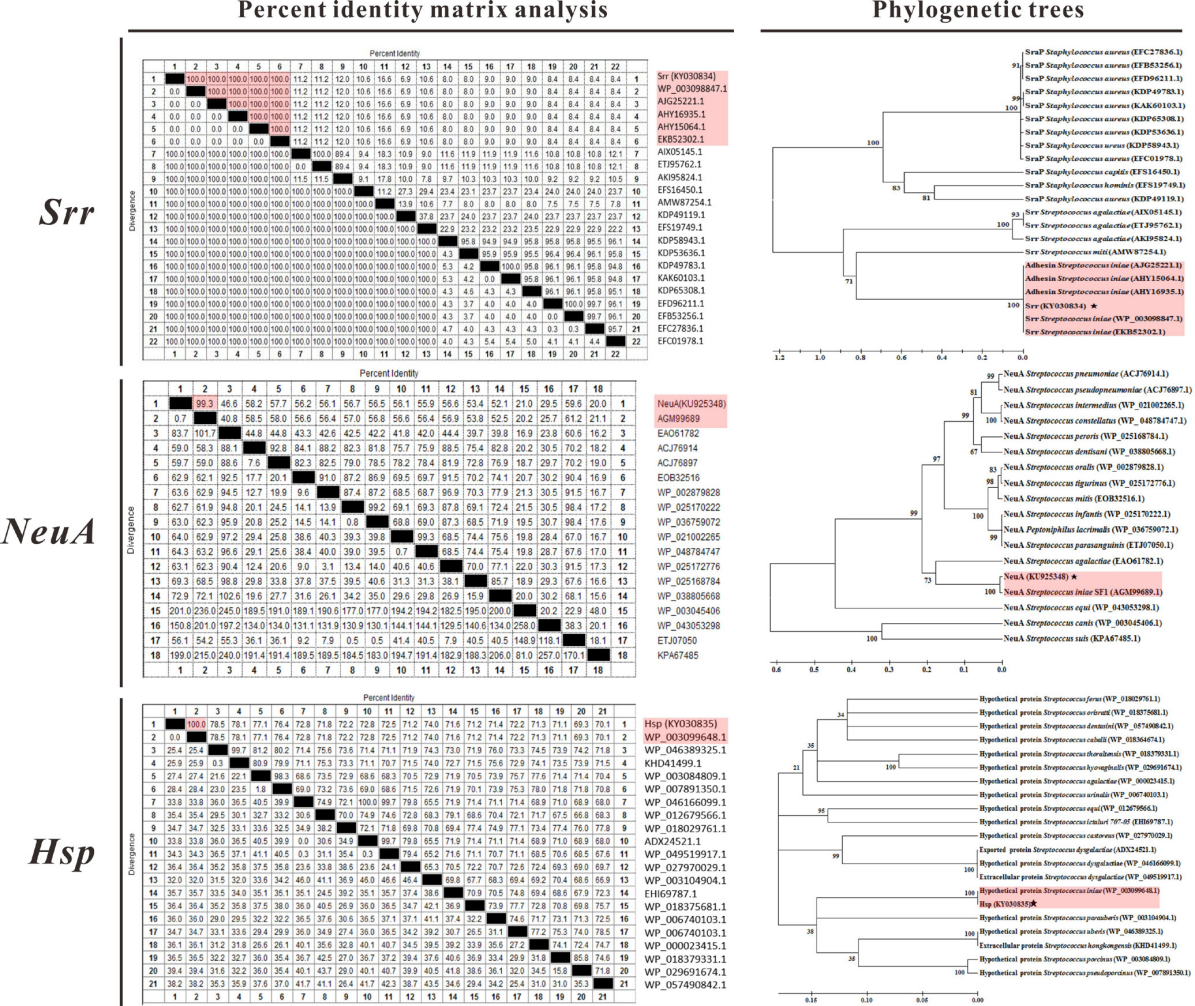
Percent identity matrix analysis and phylogenetic trees of vaccine candidate antigens with reference sequences

Multiple sequence alignment and phylogenetic analyses of three aa sequences were performed with related reference sequences using MegAlign and MEGA 5.1 software (Figure 4). The results suggested that Srr of *S. iniae* DGX07 was almost consistent with Srr or an adhesion protein of other reference *S. iniae* and clustered together with them in a phylogenetic tree, and shared low identity (6.9%-16.6%) with other *Streptococcus* spp. The NeuA in this study shared 99.3% identity and clustered together in a phylogenetic tree with *S. iniae* SF1 (AGM99689), and showed low identity ranging from 20.0% to 59.6% with other reference sequences. The Hsp of *S. iniae* DGX07 shared 100% identity with the hypothetical protein of *S. iniae* (WP_003099648) and clustered together in a phylogenetic tree, and shared 69.3%-78.5% identity with the rest of the reference sequences.

### Expression and purification of vaccine candidate antigens

**Figure 5:**
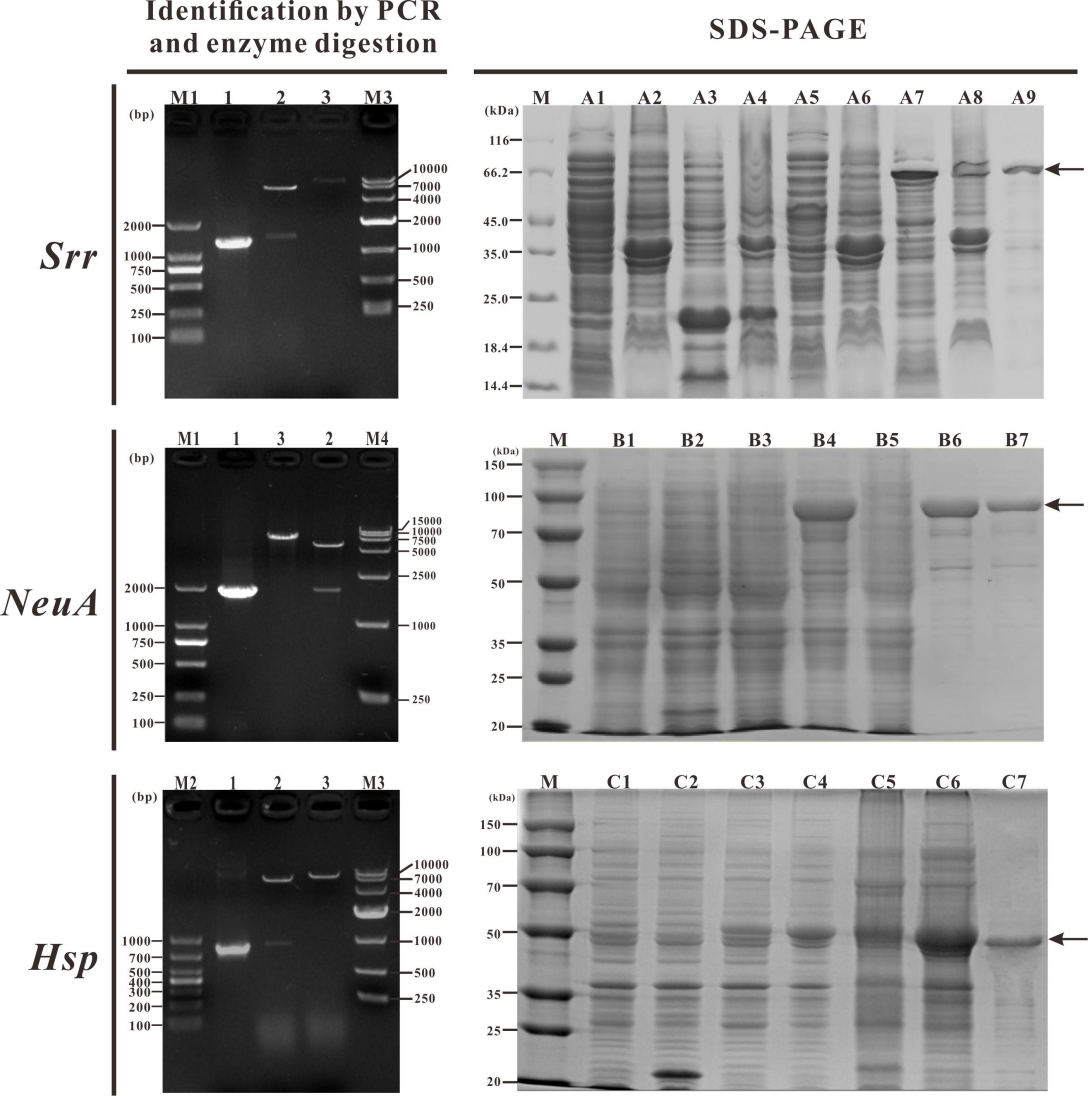
Detection of recombinant plasmids and expression and purification of recombinant proteins M1: DNA marker (DL2000); lane 1: PCR products of candidate antigen genes; lane 2: enzyme digestion of recombinant plasmids with *Bam*HI + *Xho*I; lane 3: enzyme digestion of recombinant plasmids with *Bam*HI; M2: DNA marker (DL1000); M3: DNA marker (DL10000); M4: DNA marker (DL15000). M: Protein markers. A1~A9: uninduced BL21 (pET32a) supernatant, uninduced BL21 (pET32a) sediment, induced BL21 (pET32a) supernatant, induced BL21 (pET32a) sediment, uninduced BL21 (P-Srr) supernatant, uninduced BL21 (P-Srr) sediment, induced BL21 (P-Srr) supernatant, induced BL21 (P-Srr) sediment, purification of recombinant rSrr. B1~B7: uninduced BL21 (pET32a), induced BL21 (pET32a), uninduced BL21 (P-NeuA), induced BL21 (P-NeuA), induced BL21 (P-NeuA) supernatant, induced BL21 (P-NeuA) sediment, purification of recombinant rNeuA. C1~C7: uninduced BL21 (pET32a), induced BL21 (pET32a), uninduced BL21 (P-Hsp), induced BL21 (P-Hsp), induced BL21 (P-Hsp) supernatant, induced BL21 (P-Hsp) sediment, purification of recombinant rHsp.

The plasmids expressing the recombinant proteins were extracted from recombinant *E. coli* BL21 cells and detected by PCR, enzymatic digestion, and sequencing. The sequence analysis results confirmed that the candidate antigen genes were inserted into the vector in the correct orientation and the target fragments were consistent with the expected sizes (Figure 5). The recombinant bacteria containing the plasmids P-Srr, P-NeuA, and P-Hsp were induced with IPTG and subsequently lysed by ultrasonic disruption, then the recombinant proteins were purified by His-tag Ni affinity chromatography and analyzed by SDS-PAGE. The results showed that rSrr was about 70 kDa and expressed mainly in a soluble form in the supernatant, rNeuA was about 86 kDa and expressed in an insoluble form in the sediment, and rHsp was about 51 kDa and expressed mostly in an insoluble form in the sediment (Figure 5).

### Western blot analysis of vaccine candidate antigens

The immunogenicity of recombinant proteins was further determined with rabbit anti-6-histidine antibody and rabbit antiserum to *S. iniae* by western blot analysis. The results indicated that the commercial rabbit anti-6-histidine antibody specifically detected rSrr, rNeuA, and rHsp as single bands of about 70 kDa, 86 kDa, and 51 kDa, respectively, which supported that the recombinant proteins rSrr, rNeuA, and rHsp were immunogenic. Moreover, the rabbit antiserum to *S. iniae* reacted with rSrr, rNeuA, and rHsp, respectively, indicating that these recombinant proteins retained their natural configurations.

### Specific serum antibody response

Specific antibody production was detected using an ELISA. The results showed that specific antibodies against the different candidate antigens were produced in all four treated groups, but not the control group (Figure 7). The highest antibody titers were detected at post-vaccination weeks 4, 5, 5, and 4 for fish vaccinated with iS + ISA763, rSrr + ISA763, rNeuA + ISA763, and rHsp + ISA763, respectively. Moreover, the antibody titers of the four treated groups were in the order of rSrr + ISA763 > rNeuA + ISA763 > rHsp + ISA763 > iS + ISA763. In addition, the specific antibody levels between the rSrr + ISA763 and rNeuA + ISA763 groups were significant only at week 5, while the specific antibody titers of the rHsp + ISA763 and iS + ISA763 groups were not significant except at weeks 4 and 5.

**Figure 6:**
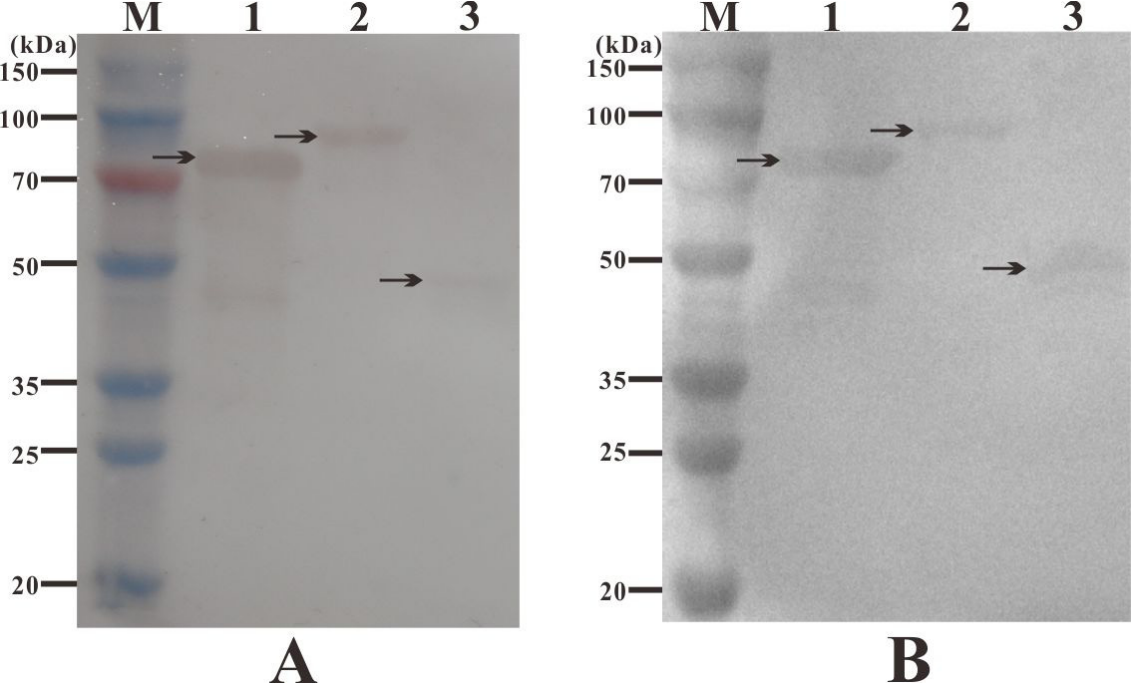
Western blot analysis of candidate antigens with rabbit anti-6-histidine antibody (**A**) and rabbit antiserum to S. iniae (**B**). M: protein marker; lane 1: recombinant protein rSrr, lane 2: recombinant protein rNeuA, lane 3: recombinant protein rHsp.

**Figure 7:**
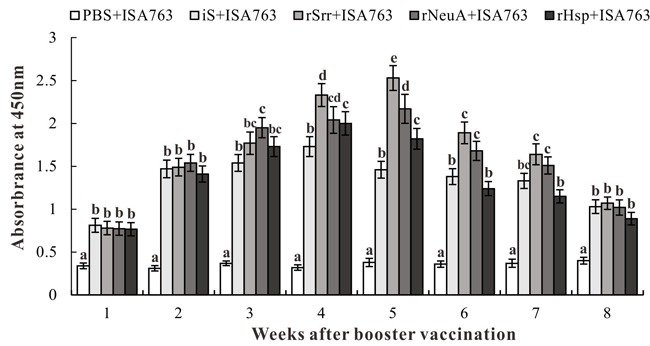
Specific antibody detection in vaccinated fish by ELISA Channel catfish were vaccinated with PBS + ISA763, iS + ISA763, rSrr + ISA763, rNeuA + ISA763 or rHsp + ISA763. Sera were collected from the fish at 1 ~ 8 weeks p.v. Data are presented as means ± SD (n = 5). Different letters indicate significant differences among groups at the same time (*p* < 0.05).

### Immunoprotective efficacy against S. iniae

**Figure 8:**
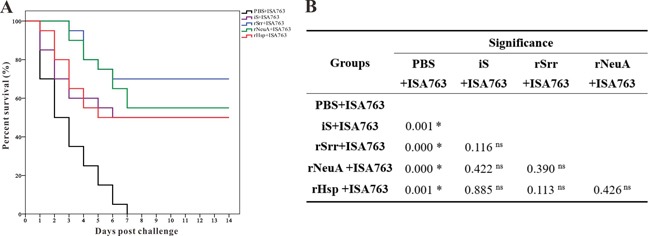
Percent survivals (Kaplan-Meier) of vaccinated fish (n=20) during the challenges tests at 4 week b.v **A**. Differences among groups were tested using the log-rank test shown in **B**.. “*”Denotes significant difference (*p* < 0.05), “ns” means not significant.

During the challenge experiment with the pathogenic *S. iniae* DGX07 at 4 weeks p.v, the accumulated survival of fish vaccinated with PBS + ISA763, iS + ISA763, rSrr + ISA763, rNeuA + ISA763, and rHsp + ISA763 was 0%, 50%, 70%, 55%, and 50%, respectively (Figure 8A), which was equal to the immunoprotective efficacies in terms of RPS, as compared with the control group. In this case, the differences in survival among the four treated groups were not all significant, but were significantly greater than that of the control group (Figure 8B).

## DISCUSSION

In this study, three candidate protective immunogens of *S. iniae* DGX07 isolated from diseased channel catfish were identified and analyzed. Molecular cloning, expression, and purification of candidate antigen genes were carried out to obtain the candidate antigens in the forms of recombinant subunit vaccines. Western blotting was performed to evaluate immunogenicity *in vitro* and channel catfish were vaccinated by intraperitoneal injection and the specific antibody titers and relative percent of survival were determined to evaluate immune protection *in vivo*. All of the experimental results indicated that compared with the inactivated *S. iniae* vaccine, the three candidate immunogens were immunoprotective in the forms of recombinant subunit vaccines for channel catfish against *S. iniae* with the immunoprotective efficacy in the order of Srr > NeuA > Hsp.

Differences in immune protection may result from the intrinsic immunogenicity and the location of the candidate immunogens. As compared with intracellular proteins, extracellular and surface proteins of bacterial pathogens have the advantage of easy recognition by the infected host and thus are more likely to serve as targets for vaccine development [29]. Actually, an abundance of extracellular or surface proteins of fish bacteria pathogens has been found to possess vaccine potential and confer immune protection [27, 29–35]. In this study, three candidate immunogens were all predicted to be located extracellularly or on the surface of *S. iniae* by the PSORTb program (data not shown). In addition, Srr contained a bacterial fibrinogen-binding adhesin SdrG domain which was anchored to the cell wall and allowed attachment of the bacteria to host tissues via specific binding [36]. NeuA, a non-viral sialidase or neuraminidase that binds to and hydrolyze terminal sialic acid residues from various glycoconjugates and plays vital roles in pathogenesis, bacterial nutrition, and cellular interactions [37], was predicted to contain a laminin G domain, which functions in signal transduction via cell-surface steroid receptors, adhesion, migration, and differentiation through mediation of cell adhesion molecules [38]. Hsp is a hypothetical secreted protein that has been detected in Firmicutes spp. and some archaebacterial [39]. Each of these three proteins was expressed successfully as a recombinant protein fused with a 6-His tag after IPTG induction and Ni-affinity purification, with the molecular weight of ~70 kDa for rSrr, ~86 kDa for rNeuA, and ~51 kDa for rHsp, respectively.

The immunogenicity of an antigen is crucial for the development of an effective subunit vaccine [28]. In the present study, the *in vitro* immunogenicity of three candidate antigens was evaluated by western blotting. The results indicated that rabbit anti-6-histidine antibody showed immunoreactivity with rSrr, rNeuA, and rHsp, respectively, suggesting that these recombinant proteins were fused correctly with the 6-His tag and supported previous predictions that all were immunogenic. Furthermore, rabbit antiserum against *S. iniae* reacted with rSrr, rNeuA, and rHsp, respectively, as the expected single bands, indicating that these recombinant proteins retained their natural configurations and had potent antigenicity [40].

To evaluate the immunogenicity or immunoprotection of candidate antigens *in vivo*, channel catfish were vaccinated intraperitoneally with the recombinant protein mixed with commercial adjuvant ISA763. Then, specific antibody levels were detected using an ELISA at p.v. weeks 1 to 8, and the PRS was determined during the challenge test with *S. iniae* DGX07 at p.v. week 4. The results showed that the antibody levels of the vaccinated fish were significantly higher than that of PBS-vaccinated fish, and subunit vaccines stimulated higher production of the specific antibody compared with inactivated *S. iniae* vaccine at p.v. weeks 4 and 5. Also, the highest antibody titers of the subunit vaccines groups were all higher than that of the inactivated vaccine group, which resulted from different types of antigens that could active both innate and adaptive (humoral and cellular) immunity. Moreover, the appearance time and the peak of specific antibody in fish vaccinated with subunit vaccines in this study was different or even brought forward, as compared with other vaccine candidate antigens against the same pathogen [14, 29, 41, 42], chiefly because of the different antigen genes and fish species, as well as different immune schedules [16].

In addition, the higher specific antibody titer might also have contributed to the higher post-challenge survival rate. Challenging vaccinated fish with a particular pathogen is an effective and direct method to evaluate the potential of a vaccine [16], and RPS is the most visual index to assess the immunoprotective effect after challenge [43]. In this study, the RPS values of fish vaccinated with iS + ISA763, rSrr + ISA763, rNeuA + ISA763, and rHsp + ISA763, were 50%, 70%, 55% and 50%, respectively, as compared with the control. Furthermore, the RPS of rSrr and rNeuA in this study were comparable to other subunit vaccines against *S. iniae*, such as the RPS of Sia10 (53.8%) in turbot [41], and the RPS of rSrr was also higher than RPS (66.9%) of mtsB in tilapia [42] and RPS (69.7%) of Sip11 in Japanese flounder [29], which was mostly due to the immunogenic differences of various candidate antigens, suggesting that the three subunit vaccines in the present study were able to offer protection against *S. iniae* infection, especially rSrr.

## MATERIALS AND METHODS

### Ethics statement

All animal experiments were reviewed and approved by the Committee of the Ethics on Animal Care and Experiments of Sichuan Agricultural University. All experimental procedures were performed in accordance with the guidelines for care and use of experimental animals of the Chinese Ministry of Science and Technology.

### Bacterial strains, plasmids, reagents and growth conditions

*S*. *iniae* DGX07 is a pathogenic isolate collected from diseased channel catfish in China and stored in our laboratory [29]. For this experiment, *S*. *iniae* DCX07 was cultured in brain-heart infusion (BHI) medium at 37°C. *Escherichia coli* DH5α and *E. coli* BL21 (DE3) cells were used as host strains for cloning and protein expression, respectively. Both cell lines were routinely grown in Luria-Bertani medium containing 100 μg/ml of ampicillin at 37°C. The plasmids pMD19-T simple (Takara Bio Inc., Otsu, Shiga, Japan) and pET32a (+) (Merck KGaA, Darmstadt, Germany) were used for T-A cloning and protein expression, respectively. Montanide™ ISA763 A VG (Seppic, Puteaux, France) was selected as a commercial adjuvant for the experiment.

### Primer design, polymerase chain reaction (PCR) amplification, and cloning of candidate antigen genes

Genomic DNA was extracted from the *S. iniae* DGX07, which was cultured to logarithmic phase in BHI broth at 37°C, using the TIANamp Bacteria DNA Kit (Tiangen, Beijing, China). The primers for genes *Srr* (serine-rich repeat glycoprotein), *NeuA* (neuraminidase A), and *Hsp* (hypothetical secreted protein) were designed based on the corresponding genes of *Streptococcus iniae* SF1, minus the signal peptide sequences, retrieved from the GenBank database (accession numbers AGM97982.1, AGM99689.1, and AGM98837.1 respectively). The target genes were amplified by PCR with the corresponding primers shown in Table 1.

**Table 1:** Primers used in this paper

Gene Name	Primers	Sequences (5′→3′)^a^	Product size (bp)	Accession Number
Srr	Srr-F	GGATCCACCATGACTGATACTGTTACA (BamHI)	1320	AGM97982.1
	Srr-R	CTCGAGTGATTGGCTTTCTCTTATTGA (XhoI)		
NeuA	NeuA-F	GGATCCATGGATTTAAGTGAACACCTG (BamHI)	1833	AGM99689.1
	NeuA-R	CTCGAGTTATTGAACAAGGCGATGAT (XhoI)		
Hsp	Hsp-F	GGATCCCAAAATGTTATTGATGAGAGC (BamHI)	867	AGM98837.1
	Hsp-R	CTCGAGTTACCCAATCAAACTTCTAAA(XhoI)		

^a^ Underlined nucleotides are restriction sites of the enzymes indicated in the brackets at the ends.

The PCR products were purified using the Agarose Gel DNA Extraction Kit (Takara) and cloned into the pMD19-T vector, followed by transformation into *E. coli* DH5α cells. The positive recombinant clones were then selected using an ampicillin/isopropyl β-D-1-thiogalactopyranoside (IPTG)/X-gal agar plate. The recombinant plasmids were identified by PCR, digested with the restriction enzymes *Bam*HI and *Xho*I, and fractionated on 1% agarose gels. DNA sequencing was conducted by TaKaRa Bio Inc., and the positive recombinant cloning plasmids were named as T-Srr, T-NeuA, and T-Hsp, respectively.

### Sequence and phylogenetic analyses

The amino acid (aa) sequences were derived from nucleotide sequences using the translate tool, while the molecular weight and isoelectric point of each peptide were calculated using ProtParam tool, both available on the ExPASy molecular biology server (http://www.expasy.org/tools) [44]. Multiple sequence alignments and the identities between each pair of aa sequences were calculated using the Clustal W method [44] using the MegAlign program [46]. A phylogenetic tree with 1, 000 bootstrap replicates was constructed with the Neighbor-Joining method using MEGA 5.1 software [47].

### Expression and purification of recombinant target proteins

The expression and purification of recombinant target proteins was conducted as described in our previous study [16]. Briefly, the plasmids T-Srr, T-NeuA, and T-Hsp were digested with the restriction enzymes *Bam*HI and *Xho*I and the resultant products were respectively inserted into the *Bam*HI/*Xho*I-digested pET32a (+) vector to construct the recombinant expression plasmids, named as P-Srr, P-NeuA, and P-Hsp, respectively. The plasmids were then transformed into *E. coli* BL21 cells and induced by adding 1.0 mM IPTG at 37°C for 4 h. The cells were then centrifuged at 8000 × g for 10 min at 4°C, suspended in sterile phosphate-buffered saline (PBS), sonicated with a Sonic Dismembrator (model 500; Thermo Fisher Scientific, Waltham, MA, USA), and examined by 12.5% sodium dodecyl sulfate polyacrylamide gel electrophoresis (SDS-PAGE). The inclusion bodies from the insoluble fractions were purified using a Ni-NTA-sefinose Column (Sangon Biotech, Shanghai, China) after dissolution in 8 M urea solution and passed through a filter with 0.22-μm pores. The refolding of the purified proteins was conducted by dialyzing gradiently from 6 M urea solution to PBS at 4°C, and then analyzed by 12.5% SDS-PAGE. The protein was quantified using the Bradford assay with bovine serum albumin (BSA) as a standard and a NanoDrop spectrophotometer (Thermo Fisher Scientific) according to the manufacturer's instructions. The purified proteins were named rSrr, rNeuA, and rHsp, respectively, and stored at −20°C.

### Western blot analysis

Western blot analysis of the recombinant proteins rSrr, rNeuA, and rHsp was performed as previously described [16]. Briefly, the purified proteins were separated by 12.5% SDS-PAGE and transferred to a PVDF membrane at 150 V for 2 h. Non-specific binding sites of the membranes were blocked by incubating for 1 h at 37°C in Tris-buffered saline with Tween 20 (TBST) containing 3% BSA. Then, the membrane was incubated with rabbit anti-*S. iniae* antibody (prepared in our lab) or rabbit anti-6-histidine antibody (Sangon Biotech) diluted 1:100 in TBST (containing 0.5% BSA) for 12 h at 4°C. After washing three times with TBST, the membrane was incubated with horseradish peroxidase (HRP)-conjugated goat-anti-rabbit immunoglobulin (Ig)G (H + L) (Sigma-Aldrich Corporation, St. Louis, MO, USA) diluted 1:5000 in TBST (containing 0.5% BSA) at 37°C for 1 h. After washing away the unbound secondary antibody, the specific antigen-bound antibody was visualized using 3, 3′-diaminobenzidine tetrahydrochloride (DAB) (Sigma-Aldrich Corporation) for 5-15 min, and terminated by rinsing with distilled water.

### Preparation of fish and vaccine

Channel catfish (50.0 ± 5.0 g) were purchased from a fish farm in Chengdu (Sichuan, China) and acclimatized in the laboratory for 2 weeks before experimental manipulation. The fish were fed a commercial diet daily and water was partly replaced every day, while maintaining the temperature at 28 ± 1°C. Before the experiments, the blood, liver, kidney, and spleen of the fish were randomly sampled. Examination of bacterial recovery indicated the presence of no bacteria and an agglutination test showed no reaction between the serum and *S. iniae* DGX07. Fish were anaesthetized with tricaine methanesulfonate (MS-222; Sigma, Beijing, China) prior to the experiments, which involved manipulations, such as injections and serum collection. The inactivated *S. iniae* vaccine (iS) was prepared as follows. *S. iniae* strain DGX07 was cultured overnight at 37°C in BHI medium, harvested by centrifugation, resuspended in sterilized PBS, and adjusted to 1 ×10^7^ CFU/ml. The bacteria were then inactivated at 37°C for 24 h with 0.2% (wt/vol) formaldehyde, followed by the confirmation of the absence of viable organisms on the BHI plate. The purified proteins rSrr, rNeuA, and rHsp were diluted in PBS to 3.0 mg/ml, respectively. To obtain PBS + ISA763, iS + ISA763, rSrr + ISA763, rNeuA + ISA763, and rHsp + ISA763, the PBS, iS, rSrr, rNeuA, and rHsp were respectively mixed with Montanide™ ISA763 A VG adjuvant (Seppic) at a ratio of 3:7 using an ultrasonic sonicator (model no. JY92-IIDN; Ningbo Scientz Biotechnology Co., Ltd., Zhejiang, China).

### Vaccination and bacterial challenge

Five hundred channel catfish were randomly divided into five groups (100 fish/group) and intraperitoneally injected with 0.2 ml of PBS + ISA763 (control group), iS + ISA763, rSrr + ISA763, rNeuA + ISA763, and rHsp + ISA763, respectively. Booster vaccinations were performed to obtain an optimal immune response with the same method and dosage at 2 weeks post-vaccination. At 4 weeks post-booster vaccination (b.v.), 20 fish from each group were randomly selected and challenged by intraperitoneal injection with 0.2 ml of *S. iniae* DGX07 that was resuspended in PBS to 6 ×10^7^ CFU/ml [16]. Mortality was monitored over a period of 14 days after the challenge and dying fish were randomly selected for examination of bacterial recovery from the liver, kidney, and spleen. Relative percent of survival (RPS) was calculated according to the following formula: RPS = [1− (% mortality of vaccinated fish / % mortality of control fish)] × 100 [48]. Serum samples of five fish in each group were collected for detection of specific antibody titers from 1 to 8 week b.v. All vaccination trials were repeated once.

### Enzyme-linked immunosorbent assay (ELISA)

Specific antibody titers for recombinant proteins were determined by ELISA as described previously with some modifications [16]. Briefly, the recombinant proteins were diluted to a concentration of 50 μg/mL in carbonate buffer (pH = 9.6). Next, 100 μL of diluted recombinant proteins was added to each well of a 96-well plate, which was incubated overnight at 4°C and then washed with PBST (0.1% Tween-20 in PBS) and blocked with 3% BSA in PBST for 2 h at 37°C. Serial 2-fold dilutions of sera were added to the wells in triplicate and the plate was subsequently incubated for 2 h at 37°C. Rabbit anti-channel catfish IgM antibody (prepared in our laboratory) (1:200) and goat-anti-rabbit IgG (H + L)-HRP (1:2000) were used as the secondary and tertiary antibodies, respectively. Color development was performed with the TMB kit (Tiangen). Absorbance was read at 450 nm using a microplate reader (Bio-Rad Laboratories, Hercules, CA, USA).

### Statistical analysis

All statistical analyses were performed using SPSS 19.0 software (SPSS Inc., Chicago, IL, USA) and the statistical significance of differences was determined using one-way analysis of variance (ANOVA). In all cases, a probability (*p*) value of < 0.05 was considered statistically significant and results are presented as means ± standard deviations (SD).
